# Endogenous Cholinergic Neurotransmission Contributes to Behavioral Sensitization to Morphine

**DOI:** 10.1371/journal.pone.0117601

**Published:** 2015-02-03

**Authors:** Dusica Bajic, Mariano Soiza-Reilly, Allegra L. Spalding, Charles B. Berde, Kathryn G. Commons

**Affiliations:** 1 Department of Anesthesiology, Perioperative and Pain Medicine, Boston Children’s Hospital, 300 Longwood Avenue, Boston, MA, 02115, United States of America; 2 Department of Anaesthesia, Harvard Medical School, 25 Shattuck St., Boston, MA, 02115, United States of America; Xi’an Jiaotong University School of Medicine, CHINA

## Abstract

Neuroplasticity in the mesolimbic dopaminergic system is critical for behavioral adaptations associated with opioid reward and addiction. These processes may be influenced by cholinergic transmission arising from the laterodorsal tegmental nucleus (LDTg), a main source of acetylcholine to mesolimbic dopaminergic neurons. To examine this possibility we asked if chronic systemic morphine administration affects expression of genes in ventral and ventrolateral periaqueductal gray at the level of the LDTg using rtPCR. Specifically, we examined gene expression changes in the area of interest using *Neurotransmitters and Receptors* PCR array between chronic morphine and saline control groups. Analysis suggested that chronic morphine administration led to changes in expression of genes associated, in part, with cholinergic neurotransmission. Furthermore, using a quantitative immunofluorescent technique, we found that chronic morphine treatment produced a significant increase in immunolabeling of the cholinergic marker (vesicular acetylcholine transporter) in neurons of the LDTg. Finally, systemic administration of the nonselective and noncompetitive neuronal nicotinic antagonist mecamylamine (0.5 or 2 mg/kg) dose-dependently blocked the expression, and to a lesser extent the development, of locomotor sensitization. The same treatment had no effect on acute morphine antinociception, antinociceptive tolerance or dependence to chronic morphine. Taken together, the results suggest that endogenous nicotinic cholinergic neurotransmission selectively contributes to behavioral sensitization to morphine and this process may, in part, involve cholinergic neurons within the LDTg.

## Introduction

Opioid analgesic abuse has evolved into a national epidemic as a significant clinical problem with devastating consequences [1]. The mesolimbic dopaminergic system originating from the ventral tegmental area (VTA), and its major projection to the anterior part of the basal forebrain, the nucleus accumbens, plays a central role in motivation, reward, and addiction of drugs of abuse, including morphine [2–6]. Specifically, high-frequency bursts of action potentials in VTA neurons are critical for reward-dependent learning [7]. Similarly, changes in the properties of excitatory synapses on VTA dopaminergic neurons (A10) [8] are considered by some the most important neural circuit changes that lead to the development of addiction [9].

However, neuroplasticity in mesolimbic circuitry following chronic administration of morphine remains under investigation. Previous work demonstrated that electrical stimulation of the cholinergic nucleus, laterodorsal tegmental nucleus (LDTg) located in the ventrolateral periaqueductal gray (PAG), promotes burst firing of VTA dopaminergic neurons and increases dopamine release in the nucleus accumbens [10,11]. Similarly, LDTg lesions attenuate dopamine efflux in nucleus accumbens [12] and striatum [13] in response to morphine. This is consistent with anatomical tracing studies, which revealed that the LDTg projects mainly to the VTA [14] and makes excitatory synapses on dopaminergic neurons projecting to the nucleus accumbens [15]. Interestingly, cholinergic neurons of LDTg/pedunculopontine tegmentum provide the only known cholinergic input to the VTA [14,16,17]. Furthermore, LDTg is located in the midbrain ventrolateral PAG, a major brainstem site of analgesic actions of systemic morphine [18,19]. Plasticity in the ventrolateral PAG appears critical not only for the development of antinociceptive tolerance [20–22] and physical dependence [23], but also for sensitization to morphine [24,25]. The latter corresponds to certain aspects of drug addiction in animal models. In our recent study [26], by using Fos immunohistochemistry, we showed that chronic systemic morphine exposure is associated with a region-specific neuroplasticity in the rat ventrolateral PAG. This effect was specific to a selective region of the ventrolateral PAG at the level of the inferior colliculus where LDTg nucleus is located. Since cholinergic neurons of the LDTg are in a position to critically influence the expression of reward and addictive behavior following chronic opioid exposure [27], we hypothesized that cholinergic neuroplasticity at the level of the LDTg can underlie, at least in part, the sensitization effects of morphine.

High-throughput studies that looked into potential gene networks associated with chronic morphine administration are limited [28]. Thus, our first goal was to analyze expression of a select panel of neurotransmitter-related genes in the ventrolateral and ventral PAG following chronic morphine treatment. We hypothesized that chronic morphine administration would be associated with gene expression changes involving, in part, cholinergic system. Furthermore, we used quantitative fluorescent immunohistochemistry to test the hypothesis that chronic morphine administration is associated with an increase of cholinergic marker, vesicular acetylcholine transporter (vAChT) at the level of the LDTg. Finally, our third goal was to examine a possible cholinergic nicotinic role on development of (1) analgesic tolerance, (2) locomotor sensitization, and (3) dependence using the nonspecific and noncompetitive neuronal nicotinic antagonist, mecamylamine. All together, presented results support the role of LDTg in enhancing reward and motor-activity function of midbrain dopamine neurons following chronic morphine administration.

## Materials and Methods

### 2.1. Animal Care and Use

Adult male Sprague-Dawley rats (250–300 g) derived from Sasco (Charles River Laboratories International, Inc., Wilmington, MA) were maintained on a 12 hours light/dark cycle. Food and water were given *ad libitum*. The Institutional Animal Care and Use Committee at Boston Children’s Hospital approved the experimental protocols for the use of vertebrate animals in this study (IACUC#12–03–2172R). Experiments were conducted according to the United States Public Health Service Policy on Humane Care and Use of Laboratory Animals and the Guide for the Care and Use of Laboratory Animals (NIH Publications No. 80–23, revised 1996). All efforts were made to minimize the number of animals used and their discomfort.

### 2.2. Quantitative Gene Expression Analysis using Polymerase Chain Reaction (PCR) Array

**Fig 1:**
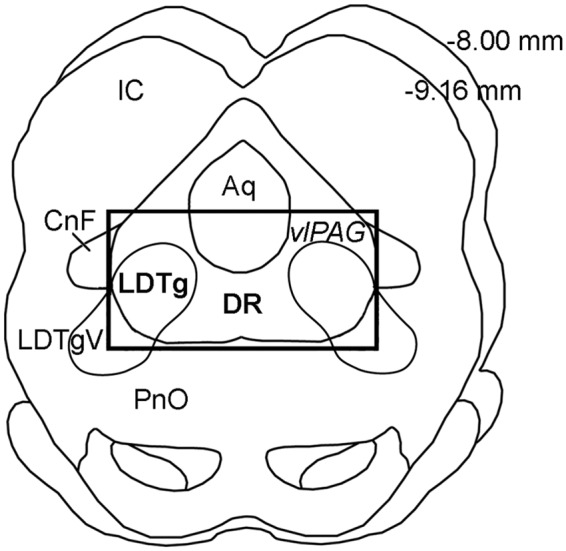
Area of Tissue Dissection for Molecular Experiments. Schematic drawing illustrates rat transverse midbrain section at the level of the inferior colliculus (**IC**). It corresponds to Plates 52–56 of the adult rat brain atlas [31]. Rectangle encloses area dissected for the isolation of the tissue used in molecular experiments. It includes laterodorsal tegmental nucleus (**LDTg**) of the ventrolateral periaqueductal gray (**vlPAG**) and dorsal raphe (**DR**). Numbers in the upper right corner illustrate distance from Bregma. *Abbreviations*: **Aq**, aqueduct (Sylvius); **CnF**, cuneiform nucleus; **LDTgV**, laterodorsal tegmental nucleus, ventral part; **PnO**, pontine reticular nucleus, oral part.

Molecular analysis included two groups that received twice-daily (9 AM and 5 PM) subcutaneous (sc) injections for 6.5 days: **(1) saline control** group that received only normal saline, and **(2) chronic morphine group** that received only morphine (10 mg/kg; Baxter Healthcare Corp., Deerfield, IL). This injection protocol was previously described [26,29]. We used a total of 40 adult rats (5 animals/group with 3–4 replicates per group). One hour following the last injection in the morning of day 7, animals were deeply anesthetized with sodium pentobarbital 100 mg/kg, intraperitoneally (ip) and decapitated. This time point was selected because antinociceptive effects following systemic administration of morphine in rats peak at around 60 min [30]. Brains were removed, and coronal tissue blocks were dissected on ice. Dissected blocks included ventrolateral and ventral PAG at the level of the inferior colliculus, where LDTg and dorsal raphe nucleus are located, respectively (Fig. 1). Tissue blocks corresponded to distances from Bregma of-7.64 to-9.16 according to the adult rat brain atlas [31]. Average weight (mg ± SD) of dissected tissue block was 26 ± 18 mg. Tissue blocks from animals of the same group (n = 5 animals/replicate/group) were collected and homogenized in 1 ml of Trizol Reagent (Life Technologies Corp., NY) for total RNA isolation using the phenol-chloroform method [32], followed by addition of 0.2 ml chloroform per 1 ml Trizol used. After incubation for 3 minutes at room temperature, samples were centrifuged at 11.000 *g* for 15 minutes at 4°C. The upper aqueous phase was transferred to another vial, and RNA was precipitated by adding 1 mcL glycogen (Invitrogen, CA) and 0.5 ml isopropyl alcohol (Sigma, St. Louis, MO) per 1 ml Trizol used. After incubation of samples for 10 minutes at room temperature, separation of RNA was accomplished by centrifugation at 11.000 *g* for 15 minutes at 4°C. Pellets containing total RNA were washed once with 1 ml 75% ethanol centrifuged at 7.500 *g* for 5 minutes at 4°C. After that, pellets containing washed RNA were dried for 3 minutes at room temperature, and then dissolved in RNAse-free water (50 μl water per 100 mg brain tissue homogenized). Samples were aliquoted and stored at-80°C. The RNA yield was evaluated in all the samples by analyzing the spectrophotometric ratios _260/280_ using a Nanodrop 2000c Spectrophotometer (ThermoScientific, West Palm Beach, FL). RNA integrity was corroborated using 2% agarose gels with ethidium bromide (3 μl/50 ml TRIS) and visualized in a UV transilluminator, showing two main bands corresponding to 18S and 28S rRNAs (data not shown). Samples containing a minimum of 5 mcg of RNA (at a concentration greater than 0.5 mg/ml) were sent on dry ice to SABiosciences (Frederick, MD) for quantitative PCR analysis using *The Rat Neurotransmitter Receptors and Regulators RT² Profiler PCR Array* (PARN-060; SABiosciences, a QIAGEN company). The Rat Neurotransmitter Receptors and Regulators PCR Array profiles the expression of 84 genes involved in modulating the biological processes of neurotransmitter biosynthesis, uptake, transport, and signaling through neurotransmitter receptors. This array contains receptors for specific neurotransmitters, such as acetylcholine, benzodiazepine, dopamine, gamma-aminobutyric acid (GABA), glutamate, serotonin, somatostatin and several neuropeptides. Genes involved in the regulation of neurotransmitter levels are included as well. Refer to Table 1 for the list of all 84 genes included in the Array and analyzed in this study. Reported genes included genes exhibiting any expression change that was significant (p<0.05) or had more than 2-fold change (100%) but was statistically marginally non-significant (p = 0.05–0.2).

**Table 1:** Gene Expression Changes Using *Neurotransmitter Receptors and Regulators PCR Array*: Chronic Morphine vs. Saline Control Groups. **Gene Expression Changes in Ventral and Ventrolateral Periaqueductal Gray with Chronic Morphine Treatment at the level of Inferior Colliculus** (for anatomical location of region of analysis, see Fig. 1). Out of 84 total genes in the *Neurotransmitter Receptors and Regulators PCR Array* (*SABiosciences*, MD), 4 genes showed statistically significant change (p<0.05), while additional 4 genes more than two-fold (100%) change that was statistically marginally non-significant (p = 0.05–0.2; two-tailed *t*-test). Total of 5 housekeeping genes were used as a reference and showed no changes between treatments. These included: *Actb*, *Hprt1*, *Ldha*, *Rpl13a*, *Rplp1*. Gene nomenclature was adopted from *SABiosciences* and corresponds to PubMed. ***Abbreviations***: ***Abat***, 4-aminobutyrate aminotransferase; ***AchE***, acetylcholinesterase; ***Anxa9***, annexin A9;***Brs3***, Bombesin-like receptor 3; ***Cckar***, cholecystokinin A receptor; ***Cckbr***, cholecystokinin B receptor; ***Chat***, choline acetyltransferase; ***Chrm1–5***, cholinergic receptor, muscarinic 1–5 (muscle); ***Chrna1–6***, cholinergic receptor, nicotinic, alpha 1–6 (neuronal); ***Chrnb1–4***, cholinergic receptor, nicotinic, beta 1; ***Chrnd***, cholinergic receptor, nicotinic, delta; ***Chrne***, cholinergic receptor, nicotinic, epsilon; ***Chrng***, cholinergic receptor, nicotinic, gamma; ***Comt***, catechol-O-methyltransferase; ***Drd1a***, dopamine receptor D1A; ***Drd2–5***, dopamine receptor D2–5; ***Gabra1–6***, gamma-aminobutyric acid (GABA) A receptor, alpha 1–6; ***Gabrb2–3***, gamma-aminobutyric acid (GABA) A receptor, beta 2–3; ***Gabrd***, gamma-aminobutyric acid (GABA) A receptor, delta; ***Gabre***, gamma-aminobutyric acid (GABA) A receptor, epsilon; ***Gabrg1–2***, gamma-aminobutyric acid (GABA) A receptor, gamma 1–1; ***Gabrp***, gamma-aminobutyric acid (GABA) A receptor, pi; ***Gabrq***, gamma-aminobutyric acid (GABA) A receptor, theta; ***Gabrr1–2***, gamma-aminobutyric acid (GABA) A receptor, rho 1–2; ***Gad1–2***, glutamate decarboxylase 1–2; ***Galr1–3***, galanin receptor 1–3; ***Glra1–3***, glycine receptor, alpha 1–3; ***Glrb***, glycine receptor, beta; ***Grpr***, gastrin releasing peptide receptor; ***Htr3a***, 5-hydroxytryptamine (serotonin) receptor 3a; ***Maoa***, monoamine oxidase A; ***Mc2r***, melanocortin 2 receptor; ***Nmur1–2***, neuromedin U receptor 1–2; ***Npffr1–2***, neuropeptides FF receptor1–2; ***Npy1r-2r*, *5r***, neuropeptide Y receptor 1–2,5; ***Ppyr1***, pancreatic polypeptide receptor 1; ***Prima1***, proline rich membrane anchor 1; ***Prlhr***, prolactin releasing hormone receptor; ***Prokr1–2***, prokineticin receptor 1–2; ***Qrfpr***, proglutamylated RFamide peptide receptor; ***Slc5a7***, solute carrier family 5 (choline transporter) member 7; ***Sstr1–5***, somatostatin receptor 1–5; ***Tacr1–3***, tachykinin receptor 1–3; ***Th***, tyrosine hydroxylase.

GENE FOLD CHANGE			
Functional Gene Grouping	#Genes Changed	#Other Genes	Total #Genes
**I. NEUROTRANSMITTER RECEPTORS**			
Cholinergic	*Chrna3* (-2.5, p<0.05)	Anxa9, Chrm1–5, Chrna1–2,4–6, Chrnb1–3, Chrnd, Chrne, Chrng	19
	*Chrnb4* (-6.5, p = 0.08)		
Inhibitory (GABA-A and Glycine)	*Gabra6* (7.4, p = 0.16)	Gabra1–5, Gabrb2–3, Gabrd, Gabre, Gabrg1–2, Gabrp, Gabrq, Gabrr2, Glra1–3, Glrb	20
	*Gabrr1* (-2.7, p<0.05)		
Monoamine	*Drd4* (-3.9, p = 0.12)	Drd1a, Drd2–3,5, Htr3a	6
Peptides	*Mc2r* (-2.9, p<0.01)	Brs3, Cckar, Cckbr, Galr1–3, Gpr83, Grpr, Nmur1–2, Npffr1–2, Npy1r-2r,5r, Ppyr1, Prlhr, Prokr1–2, Qrfpr, Sstr1–5, Tacr1–3	29
**II. REGULATION OF NEUROTRANSMITTER LEVELS**			
Biosynthesis	*Chat* (4.45, p = 0.2)	Gad1–2, Slc5a7, Th	5
Catabolism	*AChe* (7.98, p<0.05)	Abat, Comt, Maoa, Prima1	5
**TOTAL**	**8**	**76**	**84**

### 2.3. Anatomical Experiments

**Fig 2:**
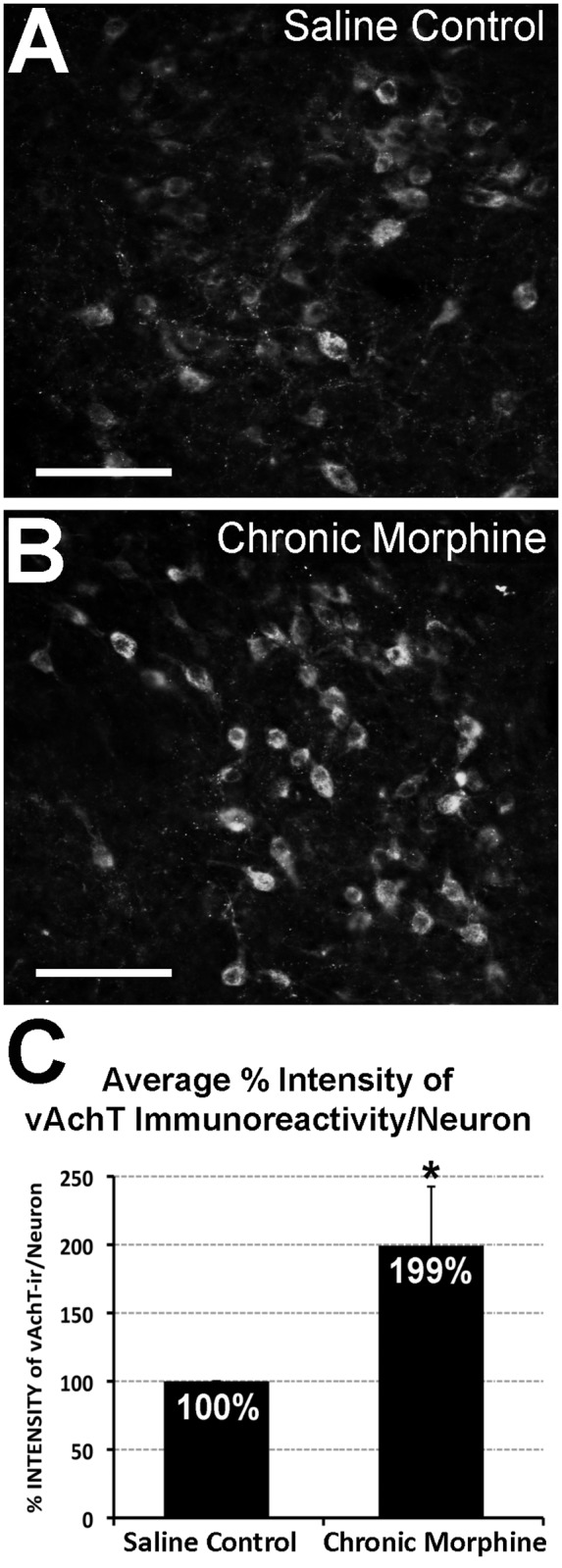
Cholinergic Neurons of the Laterodorsal Tegmental Nucleus in the Ventrolateral Periaqueductal Gray. Photomicrographs illustrate vesicular acetylcholine transporter (vAChT) immunofluorescence in the laterodorsal tegmental nucleus. Cholinergic neurons are labeled more intensely following chronic morphine treatment **(B)** in comparison to saline control group **(A)**. Graph in **Panel C** illustrates average percentage (%) intensity of vAChT immunoreactivity per individual cholinergic neuron in the laterodorsal tegmental nucleus (± SD; n = 6/group). There is a statistically significant increase (*) in intensity of vAChT immulabeling/neuron following chronic morphine administration when compared to control (99% ± 43.31; t(10) = -5.61, two-tailed p<0.001). *Scale bar* = 100 μm.

An additional set of animals (n = 6/group) was used for vesicular acetylcholine transporter (vAChT) immunohistochemistry. Animals received the same pharmacological treatment for 6.5 days as described above. Experimental groups were matched and individuals from different groups were processed in parallel. Animals were anesthetized with 100 mg/kg ip sodium pentobarbital and transcardially perfused through ascending aorta with 50 ml of NS, followed by 250 ml of 4% paraformaldehyde in 0.1M phosphate buffer (PB, pH 7.4, room temperature) on the morning of day 7. Brains were removed and stored in the same fixative solution overnight at 4°C before cryoprotection in 30% sucrose solution in 0.1M PB for at least 48 hours. Subsequently, brains were frozen and 40-micron coronal sections were cut on a freezing microtome (Leica Microsystems, Wetzlar, Germany). Free-floating sections were collected in 0.1M PB in saline and subsequently processed for vAChT immunohistochemistry. Primary (goat anti vAChT, 1:1000 dilution; Cat# ABN100; EMD Millipore, Billerica, MA) and secondary antisera (Cy3 conjugated anti-goat, 1:200 dilution; red fluorophore; Jackson ImmunoResearch Labs, Inc., West Grove, PA) were diluted in 0.1M PB with NS, 0.3% Triton X-100, 0.04% bovine serum albumin, and 0.1% sodium azide. Brain sections were incubated in primary antibodies for 2 days at 4°C, and subsequently in secondary antibodies for 1 to 2 hours at room temperature. Sections were rinsed in 0.1M PB in saline (3 times for 10 minutes) in between immunocytochemical processing. Finally, sections were rinsed in 0.1M PB in saline solution prior to mounting on slides in 0.05M PB. After drying, mounted sections were coverslipped with 90% glycerol solution. We used fluorescent/bright-field microscope (Olympus IX81; Olympus America Inc., Melville, NY, USA) equipped with a camera and digital microscopy software used for the analysis (Slidebook v4.2; Olympus). Immunolabeling with anti-vAChT antisera produced characteristic staining of the neuronal cytoplasm (Fig. 2A and B). Note that complementary sets of brain tissue were immuno-processed simultaneously and photographed with the same exposure time to minimize inter-assay variability. Specifically, bilateral pictures of the LDTg were taken uniformly with 10x magnification lens at 400 ms exposure for all brains. Average intensity labeling of vAChT immunohistochemistry per individual neuron was calculated based on analysis of intensity labeling of 5 individual neurons per picture of LDTg (total 6 pictures/brain, n = 6 brains/treatment group). Background labeling intensity (non-labeled neuropil) in each picture was subtracted from total intensity of vAChT labeling of each individual neuron that was analyzed. An observer blinded to the treatment group performed the analysis.

### 2.4. Behavioral Analysis

Additional sets of animals underwent behavioral experiments following 6.5 days of treatment with different drugs (saline sc, morphine (10mg/kg sc), and/or mecamylamine (0.5 or 2 mg/kg via intraperitoneal (ip) route; Cat#M9020; Sigma-Aldrich, St. Louis, MO). Mecamylamine is widely used as a broad-spectrum noncompetitive antagonist of neuronal nicotinic acetylcholine receptors [33]. It is clinically applicable ganglionic blocker that crosses blood-brain barrier. Mecamylamine was administered either alone or immediately following morphine administration as a separate injection. All injections were done in the home cage. Antinociceptive effects of morphine were tested using the *Hot Plate* test in the afternoon of day 7. To minimize the number of animals used, as well as to optimize the data collection, additional set of animals underwent locomotor recordings one hour after the last injection on day 7 followed by recordings of precipitated withdrawal as described below.

***2.4.1. Hot Plate Test***. We analyzed 4 pharmacological groups that received twice-daily sc injections for 6.5 days: **(1) NS control group** (n = 6), **(2) chronic morphine group** (10 mg/kg; n = 6), (**3) chronic mecamylamine group** (2 mg/kg; n = 4), and **(4) chronic morphine** (10 mg/kg) **and mecamylamine** (2 mg/kg) **group** (n = 5). Since repeated analgesic testing exaggerates the magnitude of antinociceptive tolerance in adult rats [34–36], we performed antinociceptive tolerance assessments using the *Hot-Plate Latency Test* only in the afternoon of day 7. After adaptation baseline trials, and trials following injection of NS (equivalent volume to 10 mg/kg morphine) each rat was injected with a low dose of morphine (0.1 mg/kg sc). Thirty minutes later, the rat was re-tested and injected with the next dose of morphine that was increased in a logarithmic manner using a dose range of 0.1–10 mg/kg, and increments of approximately half a log unit each time (such that each animal received 0.1, 0.3, 1, 3, and 10 mg/kg of morphine sequentially) as described before [26,29]. We used a modified *Hot Plate* test [37] (Temperature: 56°C and 12 s cutoff latency) to measure distal 2/3 hindpaw withdrawal latency in seconds. Testing began 20 minutes following the last administration of the drug. The withdrawal latency of the hindpaw of each animal was measured 3 times on both sides after each drug injection (with 10 s pause interval) and the final withdrawal latency value was averaged among 6 recordings. In none of these cases was any tissue damaged. An individual blinded to the treatment group did the behavioral testing. *Hot Plate* test data in the form of cumulative morphine dose-response curves are presented as a percentage of maximum possible effect (%MPE = (Test Latency—Baseline Latency/Cutoff Time—Baseline Latency) x100) ± SD according to the method of Harris and Pierson [38].

***2*.*4*.*2*. *Locomotor Activity Assay***. Drug sensitization is defined as an increased effect of drug following repeated doses (the opposite of drug tolerance). Classic study of sensitization relate to ‘incentive salience’, and the rewarding, addictive qualities of drugs of abuse [39]. However, there is also sensitization to the locomotor activating properties morphine, and these can be demonstrated as a consequence of repeated exposure without an intervening period of abstinence [40]. Our study of locomotor activity evaluation included another set of pharmacological groups that received twice-daily sc injections for 6.5 days: **(1) saline control** (n = 9), **(2) acute morphine group** (saline sc for 6 days and only morphine (10mg/kg sc) on the morning of day 7; n = 9), **(3) mecamylamine control** (2 mg/kg twice-daily for 6.5 days; n = 5), **(4) chronic morphine group** (10 mg/kg; n = 11). Mecamylamine effect on expression of locomotor activation was tested with **(5) chronic morphine + acute mecamylamine group** (morphine for 6 days and both morphine and mecamylamine (0.5 mg/kg or 2 mg/kg) on the morning of day 7; n = 7/group). Finally, mecamylamine effect on development of locomotor activation was evaluated by **(6) chronic morphine + chronic mecamylamine group** (morphine and mecamylamine (0.5 mg/kg or 2 mg/kg) for 6 days and only morphine on the morning of day 7; n = 7/group). All injections were done in the home cage. One hour after the last injection on day 7 (end of 6.5 days of pharmacological treatment), locomotor activity was measured in clear plexiglass cages (10”x19”x8”h; *Photobeam Activity System*, San Diego Instruments, San Diego, CA) over twelve 5-min intervals for 60 minutes. Analysis excluded the first 5-min interval to minimize differences in locomotor activity during accommodation to a new environment. Total locomotor activity data (ambulatory, fine, and rearing movements) are expressed as mean ± SEM per pharmacological group.

***2*.*4*.*3*. *Morphine Withdrawal Paradigm***. Physical dependence is manifested indirectly as a myriad of physiological disturbances and physical symptoms of withdrawal that result from abrupt morphine discontinuation or dosage reduction (spontaneous withdrawal), or as a result of opioid antagonist administration (precipitated withdrawal). The following pharmacological groups included in locomotor analysis were also used for the withdrawal analysis: **(1) saline control** (n = 10), **(2) chronic morphine group** (10 mg/kg; n = 5), **(3) chronic morphine + acute mecamylamine group** (0.5 mg/kg; n = 7 or 2 mg/kg; n = 8), and **(4) chronic morphine + chronic mecamylamine group** (0.5 mg/kg or 2 mg/kg; n = 7/group). Precipitated withdrawal was induced by a single injection of an opioid receptor antagonist, naloxone (20 mg/kg ip; N7758, Sigma-Aldrich Co., St. Louis, MO) immediately after completion of locomotor testing (2 hours after the last morphine and/or mecamylamine injections). The withdrawal was videotaped for 30 minutes. Recorded videos were analyzed and 13 behaviors were scored every 15 seconds according to the previous studies [41] by an observer blind to the treatment group. Briefly, 13 scored withdrawal behaviors corresponded to 2 different types: ‘checked signs’ and ‘graded signs’. The former are behaviors for which only the absence or presence of the behavior is evaluated and includes facial fasciculation/teeth chattering, profuse salivation, abnormal posture, erection/ejaculation, ptosis, chromodacryorrhea, and irritability. “Graded signs” are scored based on the frequency (# of events) and include escape attempts, abdominal constrictions/writhing, “wet dog” shakes, rearing, grooming, and diarrhea (# fecal boli). Modifications of the Gellert-Holtzmann method included the elimination of two behaviors (weight loss and swallowing movements), and the addition of two graded behaviors (rearing and grooming). Other modification included scoring diarrhea (#fecal boli) as a graded sign. Note that naloxone-induced withdrawal hyperalgesia (decrease in nociceptive threshold), which also refers to expression of dependence, was not performed. Withdrawal scores were expressed as individual sign means ± SD as well as the sum of all behavioral scores for each animal (total global score mean ± SD) for the 30 minutes of precipitated withdrawal.

### 2.5. Data Analysis

For gene expression analysis and average vAChT intensity labeling we used two-tailed Student’s *t*-test. For the *Hot Plate* test, locomotor testing, and morphine withdrawal behavioral assays, we used a one- or two-way analysis of variance (ANOVA) followed by either Tukey or LSD post-hoc test. Significance was established at p<0.05. All statistics were performed using IBM SPSS Statistical Software.

## Results

### 3.1. Gene expression changes in the periaqueductal gray following chronic morphine

We performed chronic opioid administration study to determine the counteradaptive molecular mechanisms that underlie neural plasticity induced by chronic morphine exposure. To determine how chronic morphine administration alters gene expression in the PAG, we analyzed gene expression differences following chronic morphine treatment in comparison to saline control using *Neurotransmitters and Receptors* PCR array. Area of analysis included ventral and ventrolateral PAG at the level of the inferior colliculus where serotonin neurons comprise dorsal raphe nucleus and cholinergic neurons comprise LDTg (Fig. 1). Molecular analysis showed that out of a total of 84 genes surveyed, only 8 genes exhibited expression changes that were either significant (p<0.05) or had more than 2-fold change (100%) but were statistically marginally non-significant (p = 0.05–0.2) (Table 1). Specifically, we report significant increase in the expression of acetylcholine esterase (***AChe***), gene encoding the enzyme responsible for the acetylcholine degradation (7.98 fold; t(4) = -2.4; p = 0.03), as well as significant decrease in gene expression following chronic morphine administration for melanocortin 2 receptor (***Mc2r***; -2.9 fold; t(4) = 5.77; p<0.01), GABAergic receptor subunit rho-1 (***Gabrr1***; -2.7 fold; t(5) = 2.02; p = 0.04), and cholinergic nicotinic alpha 3 receptor subunit (***Chrna3***; -2.5 fold, t(5) = 2.25; p = 0.03). Additional changes included a large fold increase with marginal non-significance in expression of genes encoding for GABA-A receptor, subunit alpha 6 (***Gabra6***; 7.4 fold, t(5) = -1.08; p = 0.16) and that of choline acetyltransferase (***Chat***; 4.4 fold; t(6) = -0.86; p = 0.2), the gene encoding the enzyme that synthesizes acetylcholine. Similarly, we also report a large fold decrease with marginal non-significance in gene expression of cholinergic nicotinic beta 4 receptor subunit (***Chrnb4***; -6.48 fold, t(5) = 1.66; p = 0.08) and dopamine receptor D4 (***Drd4***; -3.9 fold, t(6) = 1.33; p = 0.12). In contrast, none of the genes related to glutamatergic, noradrenergic, serotonergic, cholecystokinin, somatostatin, galanin, or glycine neurotransmission were expressed differently following chronic morphine treatment in adult rats.

### 3.2 Chronic morphine increases the expression of the cholinergic marker, vAChT, in the Laterodorsal Tegmental Nucleus

Our molecular data suggested that the cholinergic system in the region of analysis (Fig. 1) might be affected by chronic morphine administration. Specifically, 2 genes were significantly different (upregulation of ***AChe*** and downregulation of ***Chrna3***), while 2 other genes showed a large magnitude of difference, but only a trend toward significance (upregulation of ***Chat***, and downregulation of ***Chrnb4***). Our next aim was to quantify protein expression of the cholinergic marker vAChT at the level of the LDTg using a neuroanatomical approach. Specifically, we measured % intensity of vAChT fluorescent immunoreactivity per individual cholinergic neuron of the LDTg (Fig. 2). Chronic morphine treatment led to a significant increase of about 100% in vAChT immunoreactivity/neuron following chronic morphine administration when compared to control values (99% ± 43.31; t(10) = -5.61, p<0.001). This result provides additional anatomical evidence of cholinergic system alterations in the LDTg after chronic morphine administration.

### 3.3. Behavioral implications of the cholinergic system in chronic morphine effects

Our next goal was to test a possible functional role of transcriptional adaptations found in the cholinergic system upon different aspects of chronic morphine exposure. Our general working hypothesis for behavioral experiments is that nicotinic receptors are involved in chronic morphine-induced neural and behavioral plasticity. Specifically, we evaluated the effects of the selective neuronal nicotinic receptor antagonist mecamylamine on three negative behavioral aspects involved in chronic morphine treatment: antinociceptive tolerance, locomotor sensitization, and opioid dependence.

**Fig 3:**
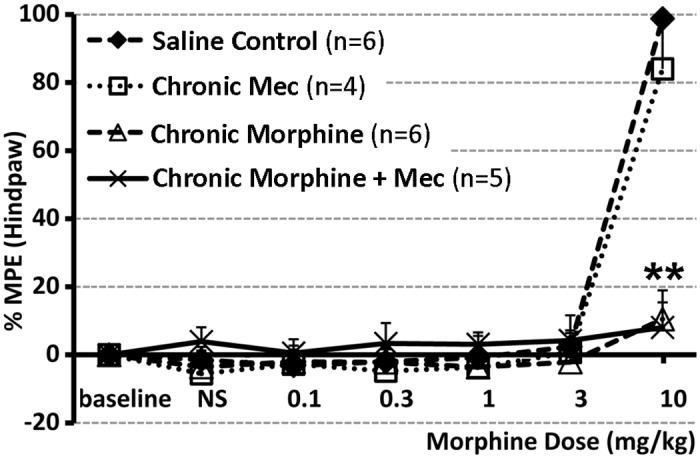
Behavioral Analysis of Rat Antinociceptive Tolerance with *Hot-Plate* Test. *Hot Plate* test was done in the afternoon on day 7 of treatment to evaluate development of antinociceptive tolerance. Chronic morphine administration (10 mg/kg sc twice-daily for 6.5 days; n = 6) was associated with development of antinociceptive tolerance in comparison to saline control group (n = 6). Chronic mecamylamine (**Mec**) administration (2 mg/kg sc twice-daily for 6.5 days; n = 4) did not change morphine antinociceptive effect. In addition, when given chronically with morphine, mecamylamine did not change development of antinociceptive tolerance (n = 5). Results are presented as a percentage of maximum possible effect (**%MPE** ± SD) according to the method of Harris and Pierson [38] to construct dose-response curves for morphine’s antinociceptive effect. Two-way ANOVA shows a significant effect of morphine (*F*(1,17) = 377.69, p<0.0001 at 10 mg/kg testing dose), while there is no significant effect of mecamylamine nor interaction effect. **, p<0.01.

***3*.*3*.*1*. *Antinociceptive Tolerance***. To evaluate the development of antinociceptive tolerance in the presence of mecamylamine (2 mg/kg), we used the *Hot Plate* test (Fig. 3). Chronic mecamylamine administration did not change antinociceptive effects of acute morphine. In addition, chronic administration of mecamylamine together with morphine did not have any effect on development of antinociceptive tolerance (Fig. 3). Specifically, two-way ANOVA shows a significant effect of morphine (*F*(1,17) = 377.69, p<0.0001 at 10 mg/kg testing dose), while there is no significant effect of mecamylamine nor interaction effect. These findings indicate that the nicotinic cholinergic system would not be involved in the development of antinociceptive tolerance induced by chronic morphine administration.

**Fig 4:**
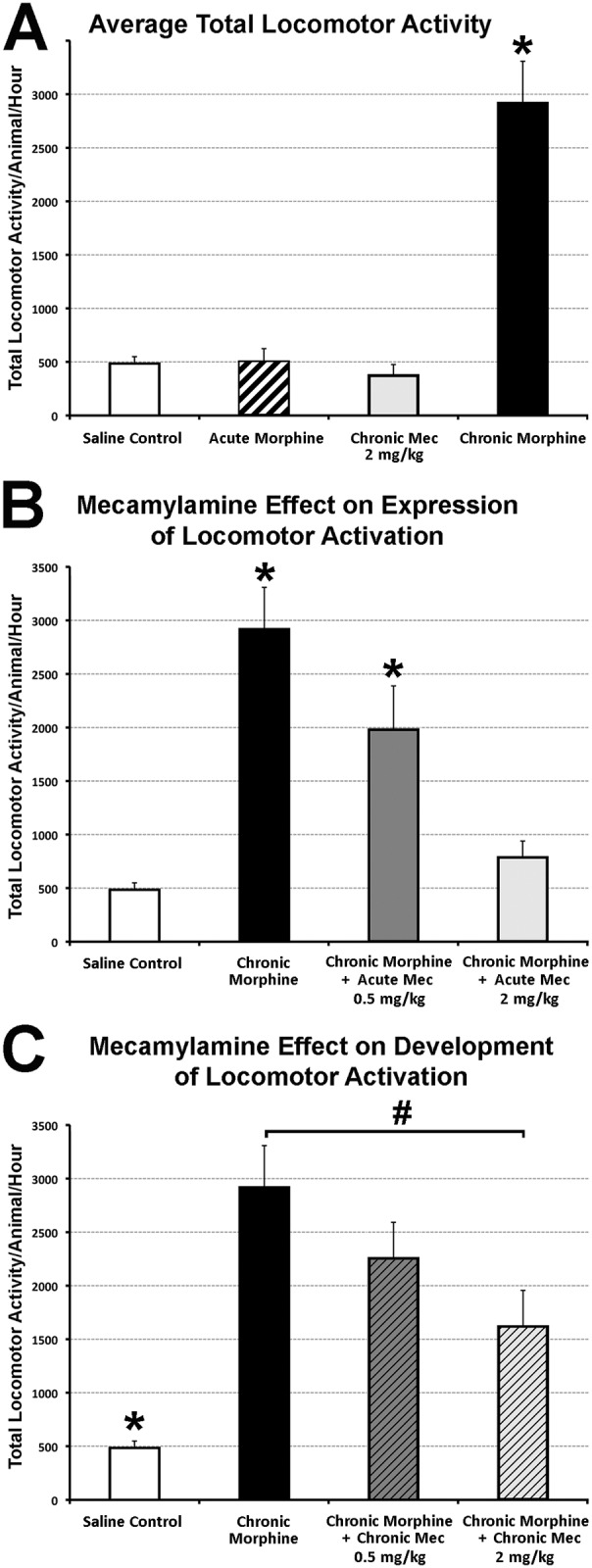
Behavioral Analysis of Rat Locomotor Activity. Graphs illustrate average total locomotor activity (ambulatory, fine, and rearing movements) ± SEM. **(A)** Chronic morphine administration (10 mg/kg sc twice-daily for 6.5 days; n = 11) was associated with locomotor sensitization when measured on day 7. It was significantly different (*F*(3,30) = 26.05, p<0.001) in comparison to saline control (n = 9; p<0.001), acute morphine group (saline sc twice-daily for 6 days and morphine 10 mg/kg sc in the morning on day 7; n = 9; p<0.001), and chronic mecamylamine (**Mec**) administration (2 mg/kg sc twice-daily for 6.5 days; n = 5; p<0.001). **Panel B** illustrates acute Mec effect on expression of locomotor activation (*F*(3,30) = 15.21, p<0.001). Mec was administered in a single dose on day 7 (0.5 or 2 mg/kg dose) to animals that were chronically treated with morphine. Although 0.5 mg/kg acute Mec dose (n = 7) statistically decreased locomotor sensitization associated with chronic morphine administration (p = 0.036), it was the 2 mg/kg dose (n = 7; p<0.001) that decreased it to the saline control level. **Panel C** illustrates chronic Mec effect on development of locomotor sensitization (*F*(3,30) = 12.37, P<0.001). Mec was administered twice daily in 0.5 or 2 mg/kg dose along morphine for 6 days. Prior to the locomotor testing in the morning of day 7, animals received only morphine. Smaller Mec dose (n = 7) had no effect, while 2 mg/kg chronic Mec administration (n = 7) significantly decreased development of locomotor sensitization in comparison to the chronic morphine group (p>0.006). However, it was still significantly higher in comparison to saline control (p<0.02). Data for saline control and chronic morphine group is the same in **A–C**. One-way ANOVA with LSD post-hoc test; *, statistically different from all other groups; #, statistical difference only between marked groups.

***3*.*3*.*2*. *Locomotor Sensitization***. We confirmed that following chronic morphine administration locomotor activity in rats increased in comparison to saline control and acute morphine treatment (Fig. 4A; *F*(3,30) = 26.05, p<0.001) validating our locomotor sensitization model. Furthermore, chronic mecamylamine administration alone had no effect on locomotor activity. In contrast, an acute dose of mecamylamine (0.5 mg/kg) on the day of testing (day 7 of morphine treatment) attenuated the expression of locomotor sensitization induced by morphine. Moreover, a higher dose of the nicotinic blocker (2 mg/kg) fully blocked the sensitizing effects of chronic morphine on locomotor activity (Fig. 4B; *F*(3,30) = 15.21, p<0.001). When we examined the effects of chronic mecamylamine on the development of locomotor sensitization induced by morphine, we found a much smaller effect of the nicotinic antagonist, only significant for the high dose tested (2 mg/kg) in comparison to chronic morphine animals (Fig. 4C; *F*(3,30) = 12.37, P<0.001). This chronic mecamylamine treatment did not fully reverse locomotor sensitization induced by morphine (Fig. 4C). Presented findings indicate that the nicotinic cholinergic system could contribute both to expression and development of locomotor sensitization induced by chronic morphine. Endogenous nicotinic transmission, however, would have a more important role in the expression than in the development phase of morphine’s sensitizing effects.

**Fig 5:**
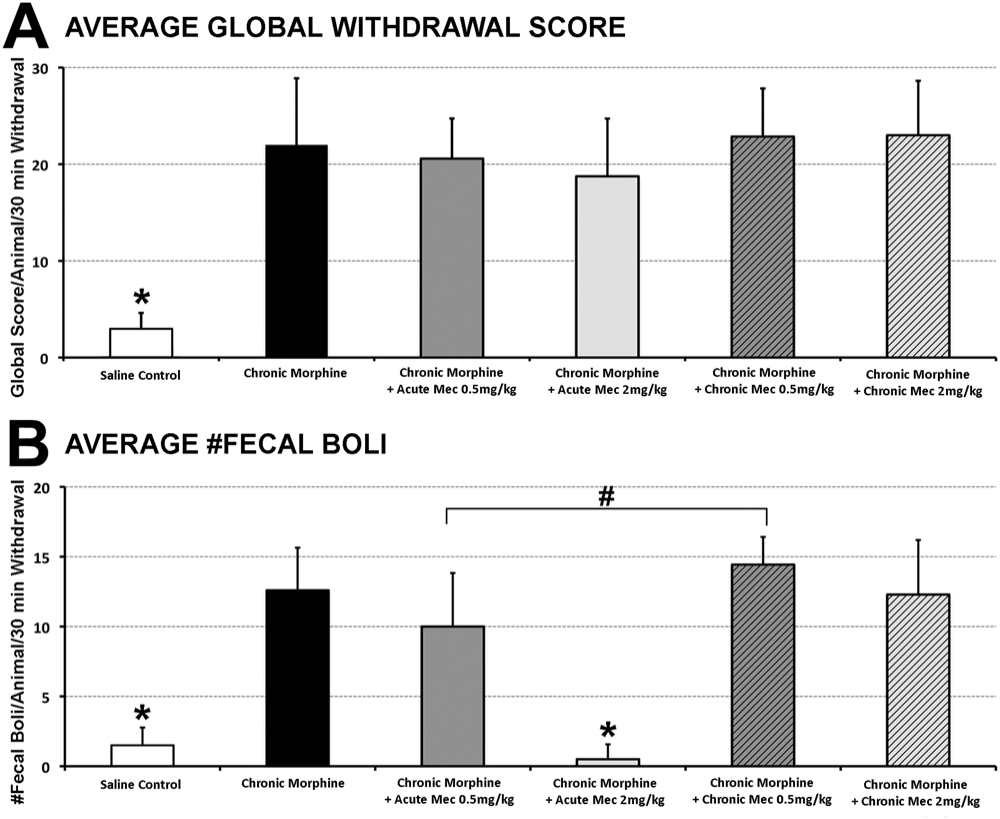
Behavioral Analysis of Rat Morphine Withdrawal. **Panel A** graph illustrates average global withdrawal score ± SD. It is significantly higher (*F*(5,38) = 22.40; p<0.001) following chronic morphine administration (n = 5) in comparison to saline control group (n = 10). However, it was not different from either acute mecamylamine (**Mec**) (n = 7 for 0.5 mg/kg dose; n = 8 for 2 mg/kg dose group), or chronic Mec groups (n = 7/group). **(B)** There were no differences in average number of individual behaviors among groups (not shown), except for the average number of fecal boli (#fecal boli/animal/30 min withdrawal ± SD; *F*(5,38) = 41.068, p<0.001). Only 2 mg/kg acute dose of Mec significantly decreased average diarrhea following chronic morphine administration (p<0.001) to a saline control level. Although average number of fecal boli is significantly decreased following 0.5 mg/kg acute dose of Mec in comparison to its chronic application (p<0.003), it was still not different from chronic MSO_4_ group. One-way ANOVA with LSD post-hoc test. *, statistically different from all other groups except each other; #, statistical difference only between marked groups.

***3*.*3*.*3*. *Opioid Dependence***. In order to further evaluate the specificity of mecamylamine’s effect on locomotor sensitization, we also evaluated physical dependence following chronic morphine administration by studying withdrawal. We induced precipitated withdrawal by administering an acute injection of the opioid antagonist naloxone following the last morphine injection on day 7. After a 30 min observation session, animals were scored for global withdrawal symptoms. Global withdrawal score means [41,42] were significantly higher in all groups that received chronic morphine (*F*(5,38) = 22.40; p<0.001). However, administration of either acute or chronic mecamylamine (0.5 or 2 mg/kg dose) had no effect on withdrawal (Fig. 5A). There were no differences in the means of individual behaviors among groups (not shown), except for the average number of fecal boli (Fig. 5B; *F*(5,38) = 41.068, p<0.001). Specifically, only 2 mg/kg acute dose of mecamylamine significantly decreased average number of fecal boli following chronic morphine administration (p<0.001) in comparison to other groups with chronic morphine administration (Fig. 5B). Although average number of fecal boli is significantly decreased following 0.5 mg/kg acute dose of Mec in comparison to its chronic application (p<0.003), it was still not different from chronic morphine group (Fig. 5B). The results show that of all the graded behaviors (weight loss, escape attempts, abdominal contractions, wet dog shakes) and checked behaviors (facial fasciculation’s or teeth chattering, swallowing movements, profuse salivation, ptosis, abnormal posture), only diarrhea (# fecal boli) was attenuated in a dose-dependent manner by an acute dose of mecamylamine.

## Discussion

In the present study, molecular analysis was done in conjunction with anatomical and behavioral studies to identify candidate neurotransmitters in the ventrolateral and ventral PAG affected by systemic chronic morphine administration. We report gene expression changes, as well as vAChT immunoreactivity changes possibly associated with an increased metabolic turnover of the cholinergic system in the area of interest, the LDTg. Furthermore, our behavioral findings support a selective role of endogenous nicotinic cholinergic neurotransmission in the expression and development of sensitizing effects of chronic morphine. On the other hand, disruption of the nicotinic cholinergic neurotransmission did not affect antinociceptive tolerance or dependence to morphine. Behavioral results indicate a clear dissociation of behavioral effects mediated by nicotinic cholinergic system following chronic morphine administration.

### 4.1. Gene Expression Changes in the Ventral and Ventrolateral PAG Following Chronic Morphine Administration

PCR is a sensitive method for detection of RNA expression levels in selected tissue. We used a pooling strategy combined with biological and technical replication to optimize this approach. In particular, pooling was used to minimize individual variation as well as technical variation caused by the dissections themselves (n = 5 rats/replicate/group). We minimized technical variation by dissecting the region of interest specifically at the level of the inferior colliculus. While our statistical approach was subject to false-positive error, relatively few differences in gene expression were detected related to treatment (morphine vs. NS control). Our study looked into the gene expression changes related to the chronic morphine paradigm. We cannot rule out that some gene transcriptional effects may not require chronic exposure. Interestingly, despite the neurochemical diversity of the region of interest, out of a total of 84 genes related to different neurotransmitter receptors and regulators, we report statistically significant changes in 4 genes (up-regulation of ***AChe***, and down-regulation of ***Mc2r***, ***Gabrr1***, and ***Chrna3***), and a large fold increase with marginal non-significance in expression of 4 additional genes (up-regulation of ***Gabra6*** and ***Chat***; down-regulation of ***Chrnb4*** *and* ***Drd4***). According to a genome-wide, high-resolution atlas of gene expression throughout the adult mouse brain (Allen Brain Atlas[43]), in situ hybridization analyses showed baseline patterned expression of mRNA of all genes (with exception of no data for *Chrnb4*) within the ventral and ventrolateral PAG, with *Chat* selectively expressed within neurons corresponding to anatomical location of cholinergic neurons comprising the LDTg [44,45]. It was also reported that alpha 7 (not included in the array) and beta 2 nicotinic acetylcholine receptor mRNAs are also co-expressed in almost all rat cholinergic cells including those of mesopontine tegmentum [46]. However, high-throughput studies that looked into potential gene networks associated with chronic morphine administration are limited. A gene expression profiling and pattern matching study in different mouse strains by Tapocik et al. [28], identified 64 genotype signature genes at the PAG that are postulated to predispose animals to morphine induced tolerance. Indeed, one of these ‘predisposition genes’ at the level of the PAG was ***Ache***. These authors also reported 81 genes (positive and negative) at the PAG that are postulated to represent potential mediators of analgesic tolerance whose expression is modulated by chronic morphine administration. Three of these ‘tolerant genes’ that included ***Gad1*** (glutamate decarboxylase 67 kDa isoform), ***Glrb*** (glycine receptor, beta subunit), and ***Cnr1*** (cannabinoid receptor 1, brain) showed up-regulations following chronic morphine treatment. None of the genes related to glutamatergic or glycine neurotransmission were detected in our study in rats. Neither our nor study by Tapocik et al. [28] reported gene changes related to noradrenergic, serotonergic, cholecystokinin, somatostatin, or galanin neurotransmission following chronic morphine treatment in rats or mice respectively. Methodological differences (PCR Array vs. microarray gene expression profiling), different animal species (rat vs. mouse), areas included in dissected tissue (ventral and ventrolateral PAG vs. PAG), and/or pharmacological treatment schedules (6 ½ twice-daily sc injections vs. three or five sc injections/3 days) could explain differences between their study and ours. Finally, future studies should include quantitative rtPCR of genes present in cholinergic neurons of the LDTg: ***Chat*** that showed large fold increase with marginal non-significance following chronic morphine administration, and genes that were not included in the array (e.g. ***vAchT***; high-affinity choline transported (***ChT***); cholinergic nicotinic receptor, alpha 7 (***Chrna7***)).

### 4.2. Neuroplasticity of the Cholinergic Neurotransmission in the LDTg Following Chronic Morphine Administration

Since our molecular data raised the possibility of increased cholinergic neurotransmission following chronic morphine administration at the level of the ventral and ventrolateral PAG, we extended PCR findings by analyzing protein expression levels of a specific cholinergic marker within neurons of the LTDg using a quantitative immunofluorescent technique. Although widely used for the last 50 years [47–49], histochemical technique to visualize acetylcholinesterase [50] was difficult to use as a quantitative measurement in the area of LDTg (not shown) because of its primary association with cholinergic terminals rather than cell bodies. Instead, we used VAChT, a proton-dependent transporter that facilitates packaging of acetylcholine into synaptic vesicles, as a useful marker for identifying both cholinergic neurons and terminals [51]. Our data show significant quantitative increase in vAChT immunolabeling in LDTg neurons at the ventrolateral PAG following administration of chronic morphine in comparison to saline control. These anatomical data support the hypothesis of an increased turnover of the cholinergic transmission following chronic morphine administration. Future studies should include quantification of LDTg protein expression with chronic morphine administration using a Western Blot technique to further support presented findings.

Cholinergic neurons of LDTg/pedunculopontine tegmentum are known to provide the only cholinergic input to the VTA [14,16,17]. Specifically, cholinergic neurons of the LDTg project mainly to the VTA [14] where they make excitatory synapses on dopaminergic neurons, which in turn project to the nucleus accumbens [15]. In fact, a major portion of cholinergic input to VTA likely involves an excitatory influence on dopaminergic mesoaccumbens neurons [52]. Furthermore, electrical stimulation of the LDTg promotes burst firing of VTA dopaminergic neurons and increases dopamine release in the nucleus accumbens [10,11]. Therefore, cholinergic neurons originating from LDTg have a pivotal role in the modulation of motivated behavior, reward pathways, and addiction (see reviews [27,53]). Demonstrated cholinergic neuroplasticity as shown by our anatomical data, could in part be implicated in the mediation of reward and addiction processes following chronic morphine administration.

### 4.3. Cholinergic Nicotinic Role in Behavioral Effects of Chronic Morphine

Presented behavioral studies confirmed that chronic morphine administration in rats [26,29] is associated with negative behavioral effects: antinociceptive tolerance (Fig. 3), locomotor sensitization (Fig. 4A), as well as opioid dependence (Fig. 5A) (see review [54]). There is a well-known dissociation in the mechanisms that underlie each of these behavioral aspects associated with chronic morphine exposure. Certainly, it is long known that the central cholinergic system is, in part, involved in opiate-induced effects [55]. Our results indicate that mecamylamine, nicotinic receptor antagonist, inhibits the neural plasticity underlying morphine locomotor sensitization but not morphine tolerance or physical dependence. Results are suggestive that nicotinic receptors are involved in mediation of specific chronic morphine induced changes in behavior, locomotor sensitization.

***4*.*3*.*1*. *Locomotor Sensitization to Morphine***. Behavioral findings support a selective role of nicotinic cholinergic neurotransmission in the expression, more than the development of sensitizing effects of chronic morphine, without affecting antinociceptive tolerance or dependence to morphine. Locomotor sensitization has been proposed to correspond to certain aspects of drug addiction such as compulsive drug-seeking behavior, and is associated with activation of the mesolimbic dopamine system (see above) [39,56,57]. This may reflect learning, however, increased locomotor response that is conditioned or context dependent is only observed if drugs of interest were administered in an environment that is similar or same to the test environment [58]. Considering that we used unpaired paradigm (animals tested in an environment that differed (unpaired) from that used for injection), recorded locomotor activity is more likely context-independent sensitization [58]. Traditionally, development of sensitization to drugs of abuse is commonly linked to the VTA, while expression of sensitization is associated with the nucleus accumbens [59]. In addition to the mesocorticolimbic system (i.e., VTA, nucleus accumbens, and medial prefrontal cortex), the ventral pallidum, hippocampus, amygdala, LDTg, and the paraventricular nucleus of the thalamus have all been suggested to play a role in the development of sensitization to drugs of abuse. We report that an acute dose of mecamylamine on the day of testing attenuated the expression, while chronic mecamylamine administration to a smaller degree decreased the development of locomotor sensitization induced by chronic morphine. These findings are supportive of different cholinergic mechanism on development and expression of behavioral sensitization to morphine (see review [59]). These results are also highly suggestive of distinct sensitivity of the involved neural networks to cholinergic modulation.

VTA was demonstrated to be critical for behavioral activation/sensitization by morphine [60–63]. It was demonstrated that nicotinic acetylcholine receptors regulate the neuronal activity in the VTA [64,65], and critically mediate the rewarding effects of morphine [66]. Activation of nicotinic acetylcholine receptors (nAChRs) in the VTA excites dopamine neurons via somatic receptors and by modulating both excitatory and inhibitory inputs [64,65,67–69]. Indeed, dopamine release in the nucleus accumbens is modulated by cholinergic activation of the VTA from brainstem cholinergic nuclei [70]. The high levels of nAChR expression in the VTA [68], along with the strong endogenous cholinergic input from the brainstem, implicated LDTg as an important cholinergic modulator of midbrain dopaminergic systems [24,27,71]. However, other brain regions with either cholinergic cells or receiving cholinergic innervation show changes in neuronal activity following morphine exposure. Morphine-induced changes in giant cholinergic interneurons in rat nucleus accumbens and striatum may serve as a model for postsynaptic plasticity involved in the long-term effects of drugs of abuse. Specifically, presynaptic opioid receptors directly modulate dopamine release in the striatum [72,73], which in turn tonically inhibits striatal cholinergic interneurons [74]. Furthermore, chronic morphine administration leads to a long-lasting augmentation of the electrically evoked acetylcholine release in slices of both nucleus accumbens and striatum [75]. Such results implicate cholinergic neurotransmission of the forebrain in the long-lasting psychomotor sensitization and enhanced vulnerability towards the acquisition of drug addiction (see review [39]). In contrast, work by Nakashini’s group investigated the role of nucleus accumbens cholinergic circuit by selectively ablating the cholinergic interneurons within the nucleus accumbens with the use of immunotoxin-mediated cell targeting techniques [76,77]. Authors reported that acetylcholine regulates the nucleus accumbens circuit jointly with dopamine but in the opposite fashion by preventing long-lasting behavioral changes of cocaine [76,77] and morphine addiction [78]. In other words, enhanced cholinergic neurotransmission in the nucleus accumbens inhibits morphine addiction. Taken together, reported opposing effects of cholinergic system on morphine addiction are possibly related to different brain areas. Future pharmacological studies (e.g. stereotaxic injections of mecamylamine directly into the VTA or selective ablation of the cholinergic neurons of the LDTg) should elucidate whether nicotinic cholinergic mechanisms that are responsible for locomotor sensitization following chronic morphine administration are mediated by cholinergic neurons originating from LDTg (as implicated by our molecular and anatomical data), as opposed to cholinergic neurons of the forebrain. In addition, these future studies might elucidate cholinergic role in development versus expression of locomotor sensitization to chronic morphine administration.

***4*.*3*.*2*. *Antinociceptive Tolerance***. Our behavioral data implicate that cholinergic nicotinic neurotransmission would not be involved in neither the antinociceptive effect of acute morphine nor the development of antinociceptive tolerance to morphine. Previous studies demonstrated that acetylcholine is involved in the manifestation of analgesia [79], as well as acute morphine effects, but had no direct role in development of morphine antinociceptive tolerance [80,81]. Although mecamylamine in the mesolimbic system antagonizes analgesia with lower doses of systemically administered morphine (5 mg/kg), this effect is “washed out” at higher doses (10 mg/kg) [82], in agreement with our findings. Together with presented molecular findings, data suggest that neuroplasticity of the cholinergic system at the level of ventral and ventrolateral PAG does not affect (1) the descending pain modulatory pathways known to be involved morphine-mediated modulation of nociception [22,83,84], or neurocircuitry critical for development of antinociceptive tolerance to morphine [20–22,85–88].

***4*.*3*.*3*. *Opioid Dependence***. Morphine dependence and withdrawal have been associated with alterations in cholinergic signaling pathways [89–91]. Our data demonstrate that chronic mecamylamine does not alter withdrawal syndrome in adult rats chronically treated with morphine. Although we did not differentiate between development and expression of opioid dependence, co-treatment of mecamylamine with chronic morphine exposure appeared to have no effect on withdrawal. These results indicate a lack of nicotinic effects in mediating morphine’s dependent state. The only individual sign that showed attenuation with acute mecamylamine administration at 2 mg/kg dose was diarrhea. Similarly to our findings, a previous study by Taraschenko et al. [92] showed that administration of mecamylamine attenuated diarrhea during withdrawal to morphine. Locus coeruleus is involved in behavioral and neurochemical changes associated with naloxone-precipitated withdrawal [93,94]. In the absence of attenuation of any other withdrawal symptoms by mecamylamine, it is highly unlikely that the neuronal nicotinic acetylcholine receptors [33] at the locus coeruleus mediate expression of diarrhea. More probable explanation is that mecamylamine reverses fecal boli excretion by blockade of densely expressed nicotinic receptors in the myenteric neurons of the gut [95] that critically regulate peristalsis [96]. In contrast to our findings, Taraschenko et al. [92] also reported attenuation in rearing and teeth chattering. Differences between their study and ours could be related to animal gender (female vs. male), or differences in morphine dosing schedule (incremental dosing [97] vs. single dose over 7 days). Finally, a recent study showed that genetic blockade of cholinergic nicotinic transmission in the medial habenula nucleus does not modify the effects of cholinergic drugs on morphine’s withdrawal [98]. Similar analysis should investigate specific administration of mecamylamine effect in the VTA. Future studies should also elucidate if mecamylamine could have a selective role in the treatment of somatic sigs of withdrawal to opioid drugs of abuse [99].

## Conclusions

Our findings contribute to a better understanding of the gene expression changes underlying systemic chronic action of morphine at the level of the PAG. Our behavioral findings support a selective role of endogenous nicotinic cholinergic neurotransmission in expression and development of locomotor sensitization to chronic morphine. Future studies should elucidate changes in cholinergic neurotransmission at the ventrolateral PAG with the selective role of LDTg in locomotor sensitization to morphine, as well as therapeutic potential of drugs acting on the nicotinic receptors in the co-treatment of addiction.

## References

[1] Cicero TJ, Inciardi JA, Munoz A (2005) Trends in abuse of Oxycontin and other opioid analgesics in the United States: 2002–2004. J Pain 6: 662–672. 16202959 10.1016/j.jpain.2005.05.004

[2] Wise RA (2004) Dopamine, learning and motivation. Nat Rev Neurosci 5: 483–494. 15152198 10.1038/nrn1406

[3] Van den Heuvel DM, Pasterkamp RJ (2008) Getting connected in the dopamine system. Prog Neurobiol 85: 75–93. 10.1016/j.pneurobio.2008.01.003 18304718

[4] Schmidt HD, Anderson SM, Famous KR, Kumaresan V, Pierce RC (2005) Anatomy and pharmacology of cocaine priming-induced reinstatement of drug seeking. Eur J Pharmacol 526: 65–76. 16321382 10.1016/j.ejphar.2005.09.068

[5] Pierce RC, Kumaresan V (2006) The mesolimbic dopamine system: the final common pathway for the reinforcing effect of drugs of abuse? Neurosci Biobehav Rev 30: 215–238. 16099045 10.1016/j.neubiorev.2005.04.016

[6] Alcaro A, Huber R, Panksepp J (2007) Behavioral functions of the mesolimbic dopaminergic system: an affective neuroethological perspective. Brain Res Rev 56: 283–321. 17905440 10.1016/j.brainresrev.2007.07.014 PMC2238694

[7] Zweifel LS, Parker JG, Lobb CJ, Rainwater A, Wall VZ, et al (2009) Disruption of NMDAR-dependent burst firing by dopamine neurons provides selective assessment of phasic dopamine-dependent behavior. Proc Natl Acad Sci U S A 106: 7281–7288. 10.1073/pnas.0813415106 19342487 PMC2678650

[8] Oades RD, Halliday GM (1987) Ventral tegmental (A10) system: neurobiology. 1. Anatomy and connectivity. Brain Res 434: 117–165. 3107759 10.1016/0165-0173(87)90011-7

[9] Luscher C, Malenka RC (2011) Drug-evoked synaptic plasticity in addiction: from molecular changes to circuit remodeling. Neuron 69: 650–663. 10.1016/j.neuron.2011.01.017 21338877 PMC4046255

[10] Forster GL, Blaha CD (2000) Laterodorsal tegmental stimulation elicits dopamine efflux in the rat nucleus accumbens by activation of acetylcholine and glutamate receptors in the ventral tegmental area. Eur J Neurosci 12: 3596–3604. 11029630 10.1046/j.1460-9568.2000.00250.x

[11] Lodge DJ, Grace AA (2006) The laterodorsal tegmentum is essential for burst firing of ventral tegmental area dopamine neurons. Proc Natl Acad Sci U S A 103: 5167–5172. 16549786 10.1073/pnas.0510715103 PMC1458812

[12] Forster GL, Falcon AJ, Miller AD, Heruc GA, Blaha CD (2002) Effects of laterodorsal tegmentum excitotoxic lesions on behavioral and dopamine responses evoked by morphine and d-amphetamine. Neuroscience 114: 817–823. 12379238 10.1016/s0306-4522(02)00365-2

[13] Miller AD, Forster GL, Metcalf KM, Blaha CD (2002) Excitotoxic lesions of the pedunculopontine differentially mediate morphine- and d-amphetamine-evoked striatal dopamine efflux and behaviors. Neuroscience 111: 351–362. 11983320 10.1016/s0306-4522(01)00595-4

[14] Cornwall J, Cooper JD, Phillipson OT (1990) Afferent and efferent connections of the laterodorsal tegmental nucleus in the rat. Brain Res Bull 25: 271–284. 1699638 10.1016/0361-9230(90)90072-8

[15] Omelchenko N, Sesack SR (2005) Laterodorsal tegmental projections to identified cell populations in the rat ventral tegmental area. J Comp Neurol 483: 217–235. 15678476 10.1002/cne.20417

[16] Oakman SA, Faris PL, Kerr PE, Cozzari C, Hartman BK (1995) Distribution of pontomesencephalic cholinergic neurons projecting to substantia nigra differs significantly from those projecting to ventral tegmental area. J Neurosci 15: 5859–5869. 7666171 10.1523/JNEUROSCI.15-09-05859.1995 PMC6577686

[17] Holmstrand EC, Sesack SR (2011) Projections from the rat pedunculopontine and laterodorsal tegmental nuclei to the anterior thalamus and ventral tegmental area arise from largely separate populations of neurons. Brain Struct Funct 216: 331–345. 10.1007/s00429-011-0320-2 21556793 PMC3255475

[18] Basbaum AI, Fields HL (1984) Endogenous pain control systems: brainstem spinal pathways and endorphin circuitry. Annu Rev Neurosci 7: 309–338. 6143527 10.1146/annurev.ne.07.030184.001521

[19] Jones SL (1992) Descending control of nociception In: Light AR, editor. The Initial Processing of Pain and its Descending Control: Spinal and Trigeminal Systems. New York: Karger pp. 203–295.

[20] Lane DA, Tortorici V, Morgan MM (2004) Behavioral and electrophysiological evidence for tolerance to continuous morphine administration into the ventrolateral periaqueductal gray. Neuroscience 125: 63–69. 15051146 10.1016/j.neuroscience.2004.01.023

[21] Morgan MM, Fossum EN, Levine CS, Ingram SL (2006) Antinociceptive tolerance revealed by cumulative intracranial microinjections of morphine into the periaqueductal gray in the rat. Pharmacol Biochem Behav 85: 214–219. 16979226 10.1016/j.pbb.2006.08.003

[22] Lane DA, Patel PA, Morgan MM (2005) Evidence for an intrinsic mechanism of antinociceptive tolerance within the ventrolateral periaqueductal gray of rats. Neuroscience 135: 227–234. 16084660 10.1016/j.neuroscience.2005.06.014

[23] Bagley EE, Hacker J, Chefer VI, Mallet C, McNally GP, et al (2011) Drug-induced GABA transporter currents enhance GABA release to induce opioid withdrawal behaviors. Nat Neurosci 14: 1548–1554. 10.1038/nn.2940 22037500

[24] Mena-Segovia J, Winn P, Bolam JP (2008) Cholinergic modulation of midbrain dopaminergic systems. Brain Res Rev 58: 265–271. 10.1016/j.brainresrev.2008.02.003 18343506

[25] Rothman RB, Blough BE, Baumann MH (2008) Dual dopamine/serotonin releasers: potential treatment agents for stimulant addiction. Exp Clin Psychopharmacol 16: 458–474. 10.1037/a0014103 19086767 PMC2683464

[26] Bajic D, Berde CB, Commons KG (2012) Periaqueductal gray neuroplasticity following chronic morphine varies with age: role of oxidative stress. Neuroscience 226: 165–177. 10.1016/j.neuroscience.2012.09.028 22999971 PMC3489988

[27] Mark GP, Shabani S, Dobbs LK, Hansen ST (2011) Cholinergic modulation of mesolimbic dopamine function and reward. Physiol Behav 104: 76–81. 10.1016/j.physbeh.2011.04.052 21549724 PMC4495915

[28] Tapocik JD, Letwin N, Mayo CL, Frank B, Luu T, et al (2009) Identification of candidate genes and gene networks specifically associated with analgesic tolerance to morphine. J Neurosci 29: 5295–5307. 10.1523/JNEUROSCI.4020-08.2009 19386926 PMC2933065

[29] Zhu H, Barr GA (2003) Ontogeny of NMDA receptor-mediated morphine tolerance in the postnatal rat. Pain 104: 437–447. 12927616 10.1016/S0304-3959(03)00051-4

[30] Johannesson T, Becker BA (1973) Morphine analgesia in rats at various ages. Acta Pharmacol Toxicol (Copenh) 33: 429–441. 4801087 10.1111/j.1600-0773.1973.tb01544.x

[31] Paxinos G, Watson C (1998) The rat brain in stereotaxic coordinates. Orlando, FL: Academic Press 10.1016/0165-0270(80)90021-7 6110810

[32] Chomczynski P, Sacchi N (1987) Single-step method of RNA isolation by acid guanidinium thiocyanate-phenol-chloroform extraction. Anal Biochem 162: 156–159. 2440339 10.1006/abio.1987.9999

[33] Papke RL, Sanberg PR, Shytle RD (2001) Analysis of mecamylamine stereoisomers on human nicotinic receptor subtypes. J Pharmacol Exp Ther 297: 646–656. 11303054

[34] Lane DA, Morgan MM (2005) Antinociceptive tolerance to morphine from repeated nociceptive testing in the rat. Brain Res 1047: 65–71. 15878767 10.1016/j.brainres.2005.04.001

[35] Plone MA, Emerich DF, Lindner MD (1996) Individual differences in the hotplate test and effects of habituation on sensitivity to morphine. Pain 66: 265–270. 8880849 10.1016/0304-3959(96)03048-5

[36] Hayes RL, Mayer DJ (1978) Morphine tolerance: is there evidence for a conditioning model? Science 200: 343–345. 635595 10.1126/science.635595

[37] Masters DB, Berde CB, Dutta SK, Griggs CT, Hu D, et al (1993) Prolonged regional nerve blockade by controlled release of local anesthetic from a biodegradable polymer matrix. Anesthesiology 79: 340–346. 8342843 10.1097/00000542-199308000-00020

[38] Harris LS, Pierson AK (1964) Some Narcotic Antagonists in the Benzomorphan Series. J Pharmacol Exp Ther 143: 141–148. 14163985

[39] Robinson TE, Berridge KC (1993) The neural basis of drug craving: an incentive-sensitization theory of addiction. Brain Res Brain Res Rev 18: 247–291. 8401595 10.1016/0165-0173(93)90013-p

[40] Norwood AP, Al-Chaer ED, Fantegrossi WE (2014) Predisposing effects of neonatal visceral pain on abuse-related effects of morphine in adult male Sprague Dawley rats. Psychopharmacology (Berl): [Epub ahead of print]. 10.1007/s00213-014-3574-6 PMC5384261 24756764

[41] Gellert VF, Holtzman SG (1978) Development and maintenance of morphine tolerance and dependence in the rat by scheduled access to morphine drinking solutions. J Pharmacol Exp Ther 205: 536–546. 566320

[42] Fdez Espejo E, Cador M, Stinus L (1995) Ethopharmacological analysis of naloxone-precipitated morphine withdrawal syndrome in rats: a newly-developed “etho-score”. Psychopharmacology (Berl) 122: 122–130. 8848527 10.1007/BF02246086

[43] Website: ©2012 Allen Institute for Brain Science. Allen Mouse Brain Atlas [Internet]. Available: http://mouse.brain-map.org/. 10.1111/cob.12003 23271062

[44] McHaffie JG, Beninato M, Stein BE, Spencer RF (1991) Postnatal development of acetylcholinesterase in, and cholinergic projections to, the cat superior colliculus. J Comp Neurol 313: 113–131. 1761749 10.1002/cne.903130109

[45] Gotti C, Guiducci S, Tedesco V, Corbioli S, Zanetti L, et al (2010) Nicotinic acetylcholine receptors in the mesolimbic pathway: primary role of ventral tegmental area alpha6beta2* receptors in mediating systemic nicotine effects on dopamine release, locomotion, and reinforcement. J Neurosci 30: 5311–5325. 10.1523/JNEUROSCI.5095-09.2010 20392953 PMC6632743

[46] Azam L, Winzer-Serhan U, Leslie FM (2003) Co-expression of alpha7 and beta2 nicotinic acetylcholine receptor subunit mRNAs within rat brain cholinergic neurons. Neuroscience 119: 965–977. 12831856 10.1016/s0306-4522(03)00220-3

[47] Koelle GB, Volle RL, Holmstedt B, Karczmar AG, O’Brien R D (1963) Anticholinesterase Agents. Science 141: 63–65. 17742888 10.1126/science.141.3575.63

[48] Godfrey DA, Williams AD, Matschinsky FM (1977) Quantitative histochemical mapping of enzymes of the cholinergic system in cat cochlear nucleus. J Histochem Cytochem 25: 397–416. 69653 10.1177/25.6.69653

[49] Silver A (1967) Cholinesterases of the central nervous system with special reference to the cerebellum. Int Rev Neurobiol 10: 57–109. 4866322 10.1016/s0074-7742(08)60151-8

[50] Hedreen JC, Bacon SJ, Price DL (1985) A modified histochemical technique to visualize acetylcholinesterase-containing axons. J Histochem Cytochem 33: 134–140. 2578498 10.1177/33.2.2578498

[51] Gilmor ML, Nash NR, Roghani A, Edwards RH, Yi H, et al (1996) Expression of the putative vesicular acetylcholine transporter in rat brain and localization in cholinergic synaptic vesicles. J Neurosci 16: 2179–2190. 8601799 10.1523/JNEUROSCI.16-07-02179.1996 PMC6578528

[52] Omelchenko N, Sesack SR (2006) Cholinergic axons in the rat ventral tegmental area synapse preferentially onto mesoaccumbens dopamine neurons. J Comp Neurol 494: 863–875. 16385486 10.1002/cne.20852 PMC2556304

[53] Lester DB, Rogers TD, Blaha CD (2010) Acetylcholine-dopamine interactions in the pathophysiology and treatment of CNS disorders. CNS Neurosci Ther 16: 137–162. 10.1111/j.1755-5949.2010.00142.x 20370804 PMC6493877

[54] Stein C (2013) Opioids, sensory systems and chronic pain. Eur J Pharmacol. 10.1016/j.ejphar.2013.10.023 23500206

[55] Frederickson RC, Pinsky C (1975) Effects of cholinergic and anticholinergic drugs and a partial cholinergic agonist on the development and expression of physical dependence on morphine in rat. J Pharmacol Exp Ther 193: 44–55. 1169318

[56] Vanderschuren LJ, Kalivas PW (2000) Alterations in dopaminergic and glutamatergic transmission in the induction and expression of behavioral sensitization: a critical review of preclinical studies. Psychopharmacology (Berl) 151: 99–120. 10972458 10.1007/s002130000493

[57] Vezina P, Leyton M (2009) Conditioned cues and the expression of stimulant sensitization in animals and humans. Neuropharmacology 56 Suppl 1: 160–168. 18657553 10.1016/j.neuropharm.2008.06.070 PMC2635339

[58] Vezina P, Giovino AA, Wise RA, Stewart J (1989) Environment-specific cross-sensitization between the locomotor activating effects of morphine and amphetamine. Pharmacol Biochem Behav 32: 581–584. 2727020 10.1016/0091-3057(89)90201-3

[59] Steketee JD, Kalivas PW (2011) Drug wanting: behavioral sensitization and relapse to drug-seeking behavior. Pharmacol Rev 63: 348–365. 10.1124/pr.109.001933 21490129 PMC3082449

[60] Vezina P, Stewart J (1984) Conditioning and place-specific sensitization of increases in activity induced by morphine in the VTA. Pharmacol Biochem Behav 20: 925–934. 6463076 10.1016/0091-3057(84)90018-2

[61] Kalivas PW, Stewart J (1991) Dopamine transmission in the initiation and expression of drug- and stress-induced sensitization of motor activity. Brain Res Brain Res Rev 16: 223–244. 1665095 10.1016/0165-0173(91)90007-u

[62] Joyce EM, Iversen SD (1979) The effect of morphine applied locally to mesencephalic dopamine cell bodies on spontaneous motor activity in the rat. Neurosci Lett 14: 207–212. 530497 10.1016/0304-3940(79)96149-4

[63] Spanagel R, Shippenberg TS (1993) Modulation of morphine-induced sensitization by endogenous kappa opioid systems in the rat. Neurosci Lett 153: 232–236. 8392157 10.1016/0304-3940(93)90329-j

[64] Mansvelder HD, Keath JR, McGehee DS (2002) Synaptic mechanisms underlie nicotine-induced excitability of brain reward areas. Neuron 33: 905–919. 11906697 10.1016/s0896-6273(02)00625-6

[65] Pidoplichko VI, DeBiasi M, Williams JT, Dani JA (1997) Nicotine activates and desensitizes midbrain dopamine neurons. Nature 390: 401–404. 9389479 10.1038/37120

[66] Rezayof A, Nazari-Serenjeh F, Zarrindast MR, Sepehri H, Delphi L (2007) Morphine-induced place preference: involvement of cholinergic receptors of the ventral tegmental area. Eur J Pharmacol 562: 92–102. 17336285 10.1016/j.ejphar.2007.01.081

[67] Mansvelder HD, McGehee DS (2000) Long-term potentiation of excitatory inputs to brain reward areas by nicotine. Neuron 27: 349–357. 10985354 10.1016/s0896-6273(00)00042-8

[68] Klink R, de Kerchove d’Exaerde A, Zoli M, Changeux JP (2001) Molecular and physiological diversity of nicotinic acetylcholine receptors in the midbrain dopaminergic nuclei. J Neurosci 21: 1452–1463. 11222635 10.1523/JNEUROSCI.21-05-01452.2001 PMC6762941

[69] Lichtensteiger W, Hefti F, Felix D, Huwyler T, Melamed E, et al (1982) Stimulation of nigrostriatal dopamine neurones by nicotine. Neuropharmacology 21: 963–968. 7145035 10.1016/0028-3908(82)90107-1

[70] Blaha CD, Allen LF, Das S, Inglis WL, Latimer MP, et al (1996) Modulation of dopamine efflux in the nucleus accumbens after cholinergic stimulation of the ventral tegmental area in intact, pedunculopontine tegmental nucleus-lesioned, and laterodorsal tegmental nucleus-lesioned rats. J Neurosci 16: 714–722. 8551354 10.1523/JNEUROSCI.16-02-00714.1996 PMC6578651

[71] Mansvelder HD, McGehee DS (2002) Cellular and synaptic mechanisms of nicotine addiction. J Neurobiol 53: 606–617. 12436424 10.1002/neu.10148

[72] Ronken E, Mulder AH, Schoffelmeer AN (1993) Interacting presynaptic kappa-opioid and GABAA receptors modulate dopamine release from rat striatal synaptosomes. J Neurochem 61: 1634–1639. 8228982 10.1111/j.1471-4159.1993.tb09797.x

[73] Schoffelmeer AN, De Vries TJ, Hogenboom F, Mulder AH (1993) Mu- and delta-opioid receptors inhibitorily linked to dopamine-sensitive adenylate cyclase in rat striatum display a selectivity profile toward endogenous opioid peptides different from that of presynaptic mu, delta and kappa receptors. J Pharmacol Exp Ther 267: 205–210. 8229747

[74] Stoof JC, Drukarch B, de Boer P, Westerink BH, Groenewegen HJ (1992) Regulation of the activity of striatal cholinergic neurons by dopamine. Neuroscience 47: 755–770. 1579210 10.1016/0306-4522(92)90027-y

[75] Tjon GH, De Vries TJ, Nestby P, Wardeh G, Mulder AH, et al (1995) Intermittent and chronic morphine treatment induces long-lasting changes in delta-opioid receptor-regulated acetylcholine release in rat striatum and nucleus accumbens. Eur J Pharmacol 283: 169–176. 7498306 10.1016/0014-2999(95)00319-g

[76] Kaneko S, Hikida T, Watanabe D, Ichinose H, Nagatsu T, et al (2000) Synaptic integration mediated by striatal cholinergic interneurons in basal ganglia function. Science 289: 633–637. 10915629 10.1126/science.289.5479.633

[77] Hikida T, Kaneko S, Isobe T, Kitabatake Y, Watanabe D, et al (2001) Increased sensitivity to cocaine by cholinergic cell ablation in nucleus accumbens. Proc Natl Acad Sci U S A 98: 13351–13354. 11606786 10.1073/pnas.231488998 PMC60874

[78] Hikida T, Kitabatake Y, Pastan I, Nakanishi S (2003) Acetylcholine enhancement in the nucleus accumbens prevents addictive behaviors of cocaine and morphine. Proc Natl Acad Sci U S A 100: 6169–6173. 12721372 10.1073/pnas.0631749100 PMC156344

[79] Hartvig P, Gillberg PG, Gordh T Jr, Post C (1989) Cholinergic mechanisms in pain and analgesia. Trends Pharmacol Sci Suppl: 75–79. 2694528

[80] Bhargava HN, Way EL (1976) Morphine tolerance and physical dependence: influence of cholinergic agonists and antagonists. Eur J Pharmacol 36: 79–88. 944135 10.1016/0014-2999(76)90259-4

[81] Bhargava VH, Saha L (2001) Cholinergic mechanism in imipramine and morphine antinociception. Indian J Pharmacol 33: 212–214.

[82] Schmidt BL, Tambeli CH, Gear RW, Levine JD (2001) Nicotine withdrawal hyperalgesia and opioid-mediated analgesia depend on nicotine receptors in nucleus accumbens. Neuroscience 106: 129–136. 11564423 10.1016/s0306-4522(01)00264-0

[83] Jensen TS, Yaksh TL (1986) Comparison of antinociceptive action of morphine in the periaqueductal gray, medial and paramedial medulla in rat. Brain Res 363: 99–113. 3004644 10.1016/0006-8993(86)90662-1

[84] Morgan MM, Whitney PK, Gold MS (1998) Immobility and flight associated with antinociception produced by activation of the ventral and lateral/dorsal regions of the rat periaqueductal gray. Brain Res 804: 159–166. 9729359 10.1016/s0006-8993(98)00669-6

[85] Jacquet YF, Lajtha A (1976) The periaqueductal gray: site of morphine analgesia and tolerance as shown by 2-way cross tolerance between systemic and intracerebral injections. Brain Res 103: 501–513. 1252940 10.1016/0006-8993(76)90448-0

[86] Siuciak JA, Advokat C (1987) Tolerance to morphine microinjections in the periaqueductal gray (PAG) induces tolerance to systemic, but not intrathecal morphine. Brain Res 424: 311–319. 3676830 10.1016/0006-8993(87)91476-4

[87] Tortorici V, Robbins CS, Morgan MM (1999) Tolerance to the antinociceptive effect of morphine microinjections into the ventral but not lateral-dorsal periaqueductal gray of the rat. Behav Neurosci 113: 833–839. 10495091 10.1037//0735-7044.113.4.833

[88] Tortorici V, Morgan MM, Vanegas H (2001) Tolerance to repeated microinjection of morphine into the periaqueductal gray is associated with changes in the behavior of off- and on-cells in the rostral ventromedial medulla of rats. Pain 89: 237–244. 11166480 10.1016/s0304-3959(00)00367-5

[89] Buccafusco JJ, Zhang LC, Shuster LC, Jonnala RR, Gattu M (2000) Prevention of precipitated withdrawal symptoms by activating central cholinergic systems during a dependence-producing schedule of morphine in rats. Brain Res 852: 76–83. 10661498 10.1016/s0006-8993(99)02197-6

[90] Crossland J, Ahmed KZ (1984) Brain acetylcholine during morphine withdrawal. Neurochem Res 9: 351–366. 6539857 10.1007/BF00963983

[91] Umberg EN, Pothos EN (2011) Neurobiology of aversive states. Physiol Behav 104: 69–75. 10.1016/j.physbeh.2011.04.045 21549137 PMC3116693

[92] Taraschenko OD, Panchal V, Maisonneuve IM, Glick SD (2005) Is antagonism of alpha3beta4 nicotinic receptors a strategy to reduce morphine dependence? Eur J Pharmacol 513: 207–218. 15862802 10.1016/j.ejphar.2005.03.005

[93] Taylor JR, Elsworth JD, Garcia EJ, Grant SJ, Roth RH, et al (1988) Clonidine infusions into the locus coeruleus attenuate behavioral and neurochemical changes associated with naloxone-precipitated withdrawal. Psychopharmacology (Berl) 96: 121–134. 3147472 10.1007/BF02431544

[94] Kimes AS, Bell JA, London ED (1990) Clonidine attenuates increased brain glucose metabolism during naloxone-precipitated morphine withdrawal. Neuroscience 34: 633–644. 2352645 10.1016/0306-4522(90)90170-9

[95] Zhou X, Ren J, Brown E, Schneider D, Caraballo-Lopez Y, et al (2002) Pharmacological properties of nicotinic acetylcholine receptors expressed by guinea pig small intestinal myenteric neurons. J Pharmacol Exp Ther 302: 889–897. 12183644 10.1124/jpet.102.033548

[96] Mandl P, Kiss JP, Vizi ES (2003) Functional neurochemical evidence for the presence of presynaptic nicotinic acetylcholine receptors at the terminal region of myenteric motoneurons: a study with epibatidine. Neurochem Res 28: 407–412. 12675123 10.1023/a:1022884231313

[97] Rho B, Glick SD (1998) Effects of 18-methoxycoronaridine on acute signs of morphine withdrawal in rats. Neuroreport 9: 1283–1285. 9631413 10.1097/00001756-199805110-00004

[98] Neugebauer NM, Einstein EB, Lopez MB, McClure-Begley TD, Mineur YS, et al (2013) Morphine dependence and withdrawal induced changes in cholinergic signaling. Pharmacol Biochem Behav 109: 77–83. 10.1016/j.pbb.2013.04.015 23651795 PMC3690589

[99] Shytle RD, Penny E, Silver AA, Goldman J, Sanberg PR (2002) Mecamylamine (Inversine): an old antihypertensive with new research directions. J Hum Hypertens 16: 453–457. 12080428 10.1038/sj.jhh.1001416

